# Quality of Life in Patients with Advanced Cancer Using the Functional Assessment of Cancer Therapy-General Assessment Tool: A Literature Review

**DOI:** 10.4021/wjon594w

**Published:** 2013-03-06

**Authors:** Marko Popovic, Nicholas Lao, Gillian Bedard, Liang Zeng, Liying Zhang, David Cella, Jennifer L. Beaumont, Nicholas Chiu, Leonard Chiu, Henry Lam, Michael Poon, Ronald Chow, Edward Chow

**Affiliations:** a Rapid Response Radiotherapy Program, Department of Radiation Oncology, Odette Cancer Centre, Sunnybrook Health Sciences Centre, University of Toronto, Toronto, Ontario, Canada; b Department of Medical Social Sciences, Northwestern University Feinberg School of Medicine, Chicago, Illinois, United States of America

**Keywords:** Advanced cancer, Quality of life, FACT-G, Treatment

## Abstract

Quality of life (QOL) has become an increasingly meaningful endpoint in advanced cancer research. Clinicians assess QOL to help them select appropriate treatment options and regimens. The present review aims to compare QOL scores of the Functional Assessment of Cancer Therapy-General Assessment Tool (FACT-G) in relation to clinical and socio-demographic features in patients with advanced cancer. A literature search in MEDLINE and EMBASE was conducted; a total of 33 studies encompassing 39 study arms were identified that reported FACT-G scores. Four statistically significant parameters were identified with respect to FACT-G scores: education, national per capita healthcare expenditures, admittance status and previous radiation therapy. A greater percentage of patients completing higher education programs were correlated to significantly better emotional well-being and global QOL. Cohorts from countries with higher national per capita healthcare expenditures had better physical well-being, social/family well-being and improved relationships with their doctors. Patient samples comprised of purely outpatients had better levels of emotional well-being and global QOL when compared to samples with a mix of outpatients and inpatients. A greater percentage of patients previously receiving radiation therapy were correlated to a better relationship with doctor score. Although limitations of the present review exist, differences in QOL scores based on socio-demographic and clinical factors are observed; certain correlations described in the present work have been described previously in the literature while others have not. Future work aimed at either determining confounding parameters or cause and effect relationships is recommended.

## Introduction

In the advanced cancer setting, patients often have limited life expectancy and present with various symptoms including pain, fatigue, confusion and depression [1]. In addition, functionality and independence is a concern for advanced cancer patients as they are often elderly. Quality of life (QOL) is a subjective, multidimensional construct that focuses on patients’ perception of their own global health status as well as nonmedical aspects of their lives [2]. Traditional cancer endpoints in clinical trials have focused on survival, however for patients with advanced disease, QOL may be more relevant [3]. In fact, the palliative/advanced cancer care settings have seen a shift of treatment intent from enhancing survival to ameliorating QOL [3].

Clinicians have historically relied on assessment tools to gauge their patients’ QOL. There are currently two widely used tools available for the general assessment of QOL in cancer patients: the European Organization for Research and Treatment of Cancer QOL Questionnaire C30 (EORTC QLQ-C30) and the Functional Assessment of Cancer Therapy-General (FACT-G).The FACT-G questionnaire, currently in its fourth version, contains four distinct subscales: physical well-being (PWB), emotional well-being (EWB), functional well-being (FWB) and social/family well-being (SWB); older versions have previously incorporated a fifth subscale-relationship with doctors (RWD). Since its inception [4], the FACT-G has been extensively validated [5].

Few studies have aimed to identify differences between socio-demographic and clinical populations in relation to their QOL scores. This information, if available, would aid in better recognizing the unique QOL burdens of a number of different patient groups. In turn, clinicians could focus management strategies based on characteristics of their patients. The purpose of the present review was to compare FACT-G scores in relation to clinical and socio-demographic features of various patient subpopulations in patients with advanced cancers.

## Methods

A literature review was conducted using the OvidSP platform in MEDLINE (1994 to 2012) and EMBASE (1994 to 2012). The search term “FACT-G” or “FACT general” was combined in a variety of ways with the following terms: “quality of life”, “palliative”, “cancer” or “advanced cancer”, “curative” or “curative treatment”, as well as the term “FACT-G.mp.”. Reference lists of articles found in the search were cross-referenced for additional pertinent articles. Only full texts were chosen for inclusion. Non-English studies and repeat data were excluded. Three coauthors independently sorted through the literature search.

Studies included in the review evaluated, in at least one study arm, QOL in advanced cancer patients at baseline using the FACT-G assessment tool; studies which utilized cancer-specific FACT modules which contained subscales of the FACT-G were also chosen for inclusion. Articles were included in the review if they reported at least one of the following pieces of information in a cohort of patients: PWB, EWB, FWB, SWB, RWD or total FACT-G score (with or without RWD). Further, only cohorts that had histologically confirmed advanced cancer (either stage III or IV carcinoma using the Roman Numeral Staging System) in greater than 50% of their cohorts were included. Data for separate study arms were recorded independently for each arm.

The following information was extracted from each article: authorship, year of publication, journal of publication, country of origin, primary cancer site, admittance status, survival time after treatment, gender, Karnofsky Performance Status (KPS) or Eastern Cooperative Oncology Group Performance Status (ECOG PS), age, prevalence of previous chemotherapy, radiation therapy, surgery or any other treatment regimens, marital status, education, disease progression and all relevant FACT-G total and subscale scores. Descriptive statistics summarized demographic and clinical characteristics; FACT-G scores were stratified by demographic and disease parameters.

### Statistical analysis

As a way of comparing FACT-G total and subscale scores in patients from different study arms and with different clinical and socio-demographic features, weighted analysis of variance (ANOVA) was conducted and PROC GLM was performed for the unbalanced data. The number of patients from each study arm was considered a weighting variable. The weighted arithmetic means and the weighted standard deviation (SD) of FACT-G scores was also calculated.

**Figure 1:**
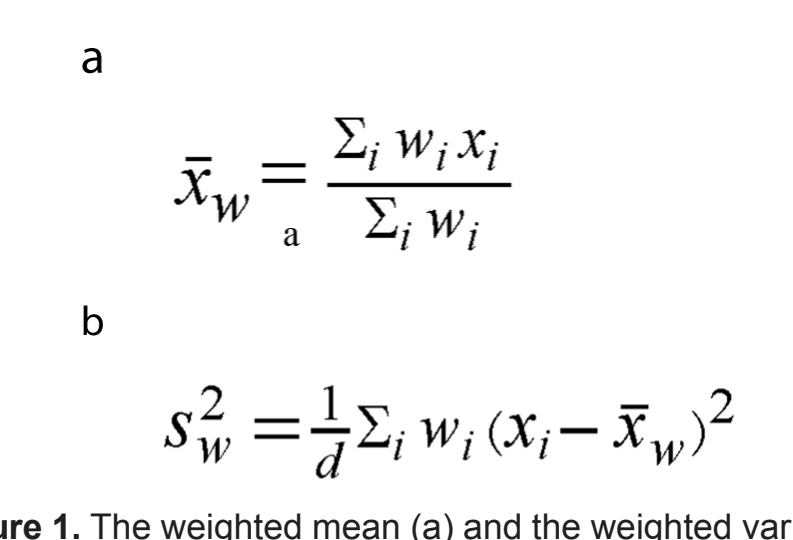
The weighted mean (a) and the weighted variance (b), wi is the weight for the ith study, xi is the ith variable value, and the variance divisor d is n-1.

The weighted mean was defined as in Figure 1a, the weighted variance was defined as in Figure 1b, where *w_i_* is the weight for the *i*th study, *x_i_* is the *i*th variable value, and the variance divisor *d* is n-1. The weighted variance is the sum of the weighted squared distance of a data value to the mean divided by the variance divisor. The weighted variance is a measure of variability.

To normalize the distribution of FACT-G total and subscale scores, a natural log-transformation was applied for each score coming from a different study arm. A P-value of less than 0.05 constituted statistical significance. All analyses were performed by Statistical Analysis of Software (SAS version 9.2 for Windows).

## Results

A total of 953 different articles were identified. Of these, 33 publications spanning a total of 39 study arms satisfied the criteria for inclusion.

### Overall FACT-G and subscale scores

For included studies using the now outdated FACT-G tool which contained the RWD subscale (n = 11), the mean overall FACT-G score was 78.63 and the median was 80.4. For studies using the updated FACT-G assessment tool without the RWD subscale (n = 22), the mean FACT-G score was 74.61 while 74.65 was the median score. Subscale scores in the included articles varied greatly across all study arms and subscales. Thirty-three included study arms had applicable information for the PWB subscale; the mean PWB subscale value reported was 19.45 while the median was 20.10. In study arms reporting mean FWB data (n = 33), the mean FWB score was 16.07 and the median was 16.90. Arms that included EWB information (n = 35) had mean and median scores of 15.82 and 16.70, respectively. The 32 study arms that disclosed SWB scores had a mean of 21.29 and a median of 22.25. Finally, only six study arms reported scores for the now defunct RWD subscale; the mean RWD score was 7.18 and the median was 7.25.

### Study characteristics

In total, there were four different objectives in the included studies (Table 1a). The aim of 13 studies was to assess the QOL of their patient populations [6-18]. The objective of another 10 studies was to establish the reliability or validity of external assessment tools by using the already robust FACT-G tool [19-28]. The approach of seven studies was to either determine correlation between QOL and other clinical factors or to examine the determinants of QOL [29-35]. Finally, three studies assessed the reliability and validity of the FACT-G instrument [36-38].

**Table 1a:** Socio-demographic and Clinical Parameters of Included Patients at Baseline

Parameter	No. (%)
Purpose of Study (n = 33)	
Assess Quality of Life	13 (39.4%)
Establish Reliability of External Assessment Tool	10 (30.3%)
Determine Determinants of Quality of Life	7 (21.2%)
Establish Reliability of the FACT-G	3 (9.1%)
Primary Cancer (n = 33)	
Heterogeneous	12 (36.4%)
Lung	7 (21.2%)
Head and Neck	4 (12.1%)
Prostate	2 (6.1%)
Breast	2 (6.1%)
Other	6 (18.2%)
Country of Study (n = 32)	
United States	17 (51.5%)
Canada	3 (9.1%)
Japan	3 (9.1%)
China	3 (9.1%)
Australia	1 (3.0%)
India	1 (3.0%)
Philippines	1 (3.0%)
South Korea	1 (3.0%)
Sweden	1 (3.0%)
Uruguay	1 (3.0%)

Of the 33 included studies, only 12 looked at samples with heterogeneous primary cancers [6, 8, 20, 26, 28, 29, 31-34, 37, 38]. Of the 21 studies that looked at patients with only one primary cancer, seven looked at patients with lung cancer [12-14, 23, 25, 30, 36], four studies observed patients with only head and neck cancers [9, 16, 24, 27], two looked at only prostate patients [7, 11], another two focused on breast patients [18, 21], those with only gynecologic cancers were examined in two studies [17, 35], one study focused on gastric carcinoma patients[19], one study analyzed patients with hepatocellular carcinoma [15], another examined renal cell carcinoma patients [22] and a further study enrolled patients with pancreatic cancer [10].

### Socio-demographic features

Included studies came from medical centres in 10 countries (Table 1a, b). Seventeen included publications came from the United States, reflecting the predominance of the FACT tools over EORTC instruments in this country. Three studies came from Canada, three from Japan, three from China, and one each from Australia, India, the Philippines, South Korea, Sweden and Uruguay. One study did not disclose its country of origin.

**Table 1b:** Socio-demographic and Clinical Parameters of Included Patients at Baseline

Parameter	No. (%)
Gender (n = 38)	
≤ 50% Male	16 (42.1%)
> 50% Male	22 (57.9%)
Mean Age, years (n = 25)	
< 49	2 (8.0%)
50 - 59	12 (48.0%)
60 - 69	11 (44.0%)
Marital Status (n = 14)	
50-70% Married	5 (35.7%)
> 70% Married	9 (64.3%)
High School Education (n = 10)	
≤ 50% Completed High School	4 (40.0%)
> 50% Completed High School	6 (60.0%)
College/University Education (n = 11)	
≤ 50% Completed College/University	8 (72.7%)
> 50% Completed College/University	3 (27.3%)
Admittance Status (n = 17)	
Purely Inpatients	1 (5.9%)
Purely Outpatients	11 (64.7%)
Both Inpatients and Outpatients	5 (29.4%)
Median Survival (n = 11)	
≤ Six Months	4 (36.4%)
> Six Months	7 (63.6%)
Median ECOG Performance Status (n = 15)	
0	2 (13.3%)
1	12 (80.0%)
2	1 (6.7%)
Previous Chemotherapy (n = 26)	
≤ 50%	6 (23.1%)
> 50%	20 (76.9%)
Previous Radiation Therapy (n = 16)	
≤ 50%	11 (68.8%)
> 50%	5 (31.3%)
Previous Surgery (n = 14)	
≤ 50%	6 (42.9%)
> 50%	8 (57.1%)

Of the 39 study arms included, seven had cohorts with less than 25% male patients, nine had patients that were between 25-50% male, 14 had patients that were between 50-75% male and eight had patients that had greater than 75% male patients. One study did not report any gender information of its cohort.

Of the 25 study arms that disclosed mean ages for its patient populations, two had populations younger than 49 years of age, 12 had populations between 50 to 59 years and 11 had populations between 60 to 69 years. Fourteen study arms reported median ages of its patients; four, seven and three arms had populations between 50 - 59, 60 - 69 and 70 - 79 years of age, respectively.

A total of 14 study arms disclosed the marital status of their patients. Of these, five study arms had between 50% and 70% of patients being married while nine arms had greater than 70% of their patient sample being married. There were no studies in the literature that reported patient samples that had less than 50% of patients married.

Only a few studies reported education characteristics of their patient samples. Ten study arms disclosed whether their patients completed high school; four arms had less than 50% of patients completing high school while another six arms had greater than 50% of patients completing high school. Of the 11 studies that reported on whether their patients completed college or university, eight had less than 50% of patients who completed college/university while another three had greater than 50% of their patient sample completing college or university.

### Clinical features

The present review considered patient samples which were mostly heterogeneous for stages of cancer malignancy (Table 1a, b). A considerable number of study arms (n = 27) reported on whether any of their patients had stage I cancer; 17 arms described that none of its patients had stage I malignancy while another 10 studies had between 0.6% and 30% of its patients having stage I disease. Twenty eight study arms reported on whether any patients in its sample had stage II disease; 16 arms had none of patients with stage II disease while another 12 study arms had anywhere between 1.70% to 44% of its patients with histologically confirmed stage II disease. Presence of stage III disease was available in 25 study arms of which 15 arms had anywhere between 0-24% of its patients with stage III malignancy while another 10 studies had greater than 25% of its patient population with stage III disease (range: 30% to 100%). Stage IV malignancy data was available in 26 study arms. Half of these arms (n = 13) had less than 50% of its patient sample with stage IV cancer (range: 0% to 49.30%) and half (n = 13) had greater than 50% of its patients with stage IV disease (range: 62% to 100%).

Eleven of the identified studies had purely outpatient patient populations, five had a mix of inpatients and outpatients, one article had only inpatients while 16 studies did not disclose whether their patients were inpatients or outpatients.

A considerable number of study arms (n = 29) did not report the median survival of its patient cohorts. Of those that did (n = 11), 36.4% (n = 4) had patients that had a median survival time of less than six months, while 63.6% (n = 7) had cohorts which had a median survival time greater than six months.

With respect to performance status, only 13 and 15 study arms reported mean and median ECOG PS, respectively. Of these, two study arms had patients with mean ECOG PS between 0 to 0.5, eight arms had mean ECOG scores between 0.5 to 1 and a further three arms had mean performance scores between 1 and 1.5. Two, 12 and one study arms had median ECOG scores of 0, 1 and 2, respectively. Only two study arms reported mean KPS and only four arms reported median KPS scores; due to the low number of arms, these values were not subject to analysis.

With respect to past treatments of patients before the FACT-G was administered, 26 study arms described the incidence of past chemotherapy, 16 study arms had information regarding past radiation therapy and 14 arms gave descriptions of past surgery. Of the 26 study arms reporting past chemotherapy frequency, three had less than 25% of their patients previously receiving chemotherapy, three had between 25% and 50% of patients receiving chemotherapy, five had between 50% and 75% of patients receiving chemotherapy and a large number of arms (n = 15) had greater than 75% of patients receiving chemotherapy. Of the 16 study arms that had information on past radiation therapy, seven had less than 25% of patients receiving prior radiation therapy, four had between 25% and 50% of patients receiving radiation therapy, two had between 50% and 75% of patients receiving radiation therapy and three had greater than 75% of patients receiving radiation therapy. Four, two, three and five study arms that reported whether their patients received prior surgery had less than 25% of patients receiving prior surgery, between 25% and 50% of patients receiving surgery, between 50% and 75% of patients receiving surgery and greater than 75% of patients receiving surgery, respectively. Only three study arms reported that hormonal therapy was administered to at least some of their patients before the FACT-G was administered.

### Assessment of QOL in various patient populations

Studies were grouped by country of origin using per capita total health expenditures data published in a 2011 report by the World Health Organization [39]. Two groups were established-group one contained countries which had per capita health expenditures of less than $2,000 US while studies which came from countries with greater than $2,000 US in per capita expenditures were sorted into a second group. Studies from China, India, the Philippines and South Korea were sorted into group one [6, 13, 15, 24, 25, 35, 37] while articles from the United States, Canada, Sweden, Japan and Australia were sorted into the second group [7-12, 14, 16, 17, 19-23, 26-34, 36, 38]. It was found that patients from countries with low per capita health expenditures had significantly lower levels of physical well-being (weighted mean: 16.87 versus 19.93; P = 0.0110), social/family well-being (mean: 18.51 versus 22.06; P = 0.0164) and relationship with doctors (mean: 6.60 versus 7.27; P = 0.0111) (Table 2); total FACT-G score (including or excluding the relationship with doctor subscale) was not statistically significant (P = 0.5964, P = 0.1652, respectively). In addition, FWB and EWB did not reach statistical significance.

**Table 2:** Weighted Analysis of Variance of Statistically Significant Clinical and Socio-Demographic Parameters in Relation to FACT-G Total and Subscale Scores

FACT-G	Categories	Sum of Weight	Weighted Mean (SD)		P-value
Physical Well-being					
Country	High Healthcare Cost	3325	19.93 (28.47)		0.0110
	Low Healthcare Cost	1039	16.87 (43.54)		
Emotional Well-being					
Admittance Status	Mixed	889	13.30 (46.28)		0.0434
	Outpatient	1816	16.59 (27.35)		
% Completing College/University	≤ 30%	736	15.10 (28.15)		0.0398
	> 30%	717	17.66 (8.06)		
Social/Family Well-being					
Country	High Healthcare Cost	3446	22.06 (31.17)		0.0164
	Low Healthcare Cost	834	18.51 (35.72)		
	> 75%	1532	20.73 (41.45)		
FACT-G Total Scale (Excluding Relationship with Doctor Subscale)					
Admittance Status	Mixed	889	62.60 (135.80)		0.0071
	Outpatient	1945	75.58 (63.68)		
% Completing College/University	≤ 30%	1126	69.60 (103.36)		0.0477
	> 30%	717	78.49 (41.66)		
Relationship with Doctor*					
Country	High Healthcare Cost	419	7.27 (1.21)		0.0111
	Low Healthcare Cost	66	6.60 (NA)		
% Receiving Previous Radiation Therapy	≤ 25%	222	7.30 (0.00)		< 0.0001
	> 25%	47	7.10 (NA)		

* There are only 6 study arms with available information for the relationship with doctors subscale. NA: not available for calculation. SD: standard deviation.

As only one study included in the review had a patient population comprised of purely inpatients [31] with a relatively low sample size (n = 90), weighted analysis of variance comparing inpatients and outpatients would be rather fruitless. Instead, studies were grouped based on whether they solely analyzed outpatient populations or whether they contained a mix of outpatients and inpatients. It was found that studies with purely outpatient populations [9, 13, 15, 20, 25-27, 29, 30, 35, 38] had better emotional well-being (mean: 16.59 versus 13.30; P = 0.0434) as well as significantly higher global QOL (mean for total FACT-G score without RWD: 75.58 versus 62.60; P = 0.0071) when compared with studies with a mix of outpatients and inpatients [19, 32, 33, 36, 37] (Table 2). All other FACT-G scores comparing the two groups did not reach statistical significance.

Certain studies disclosed the percentage of patients who had completed college or university and were either allocated into a group comprised of cohorts with ≤ 30% of patients completing college/university or a group made up of cohorts with > 30% of patients completing college/university. It was found that emotional well-being was higher in patient populations which had > 30% of patients completing post-secondary education (mean: 17.66 versus 15.10; P = 0.0398). Total FACT-G scores without RWD were also higher in this group than in patient populations with ≤ 30% of patients completing post-secondary programs (mean: 78.49 versus 69.60; P = 0.0477). Although comparisons between PWB, FWB and SWB did not reach statistical significance, it is important to note that all three of these subscales had p-values less than 2% over the P = 0.05 threshold needed to constitute significance (PWB mean: 20.42 versus 17.58; P = 0.0658); (FWB mean: 18.02 versus 15.50; P = 0.0578); (SWB mean: 22.43 versus 19.47; P = 0.0542).

One final statistically significant finding was made when cohorts were grouped based on the percentage of patients receiving previous radiation therapy. For the RWD subscale, it was found that cohorts that had ≤ 25% of patients receiving previous radiation therapy had significantly higher RWD scores when compared with cohorts that had > 25% of patients receiving previous radiation therapy (mean: 7.30 versus 7.10; P < 0.0001).

Weighted analysis of variance was conducted on five other socio-demographic/clinical parameters: heterogeneity of primary cancers, median survival, mean age and percentage of patients previously receiving chemotherapy and surgery. No statistically significant results were found for any of the FACT-G total and subscale scores for these five parameters. Other clinical or socio-demographic features were not analyzed because involved cohorts had limited sample sizes making direct statistical comparison challenging.

## Discussion

Previously, studies in the literature have not made the identification of differences among various socio-demographic and clinical populations with respect to QOL scores a focus of QOL research. If this information was available it could help with focusing management strategies based on the medical and socio-demographic information of the patient. Thus, the present review was undertaken to compare QOL scores in relation to clinical and socio-demographic characteristics of various patient subpopulations in patients with advanced cancer. Statistically significant results were obtained for four separate parameters: education, healthcare expenditures, patient status and incidence of previous radiation therapy.

When countries were grouped by their levels of total health expenditures per capita, it was found that countries with relatively lower per capita health expenditures had lower QOL in the domains of physical well-being (weighted mean: 16.87 versus 19.93; P = 0.0110), social/family well-being (mean: 18.51 versus 22.06; P = 0.0164) and relationship with doctors (mean: 6.60 versus 7.27; P = 0.0111). Due to the higher standard of care and timely access to treatment found more readily in countries with higher per capita health expenditures, patients receiving treatment in these countries may have access to more sophisticated and targeted treatment regimens which improve relative PWB; similarly, they may be exposed to a more extensive support network of health care professionals which may be the reason for higher relative RWD values [40]. In addition, although no published evidence exists, it is hypothesized that countries with higher health expenditures may also promote and support more educational and awareness programs aimed at discussing the issues faced by the advanced cancer population with the friends and families of these patients - this may be why patients in these areas reported higher SWB scores.

With respect to patient admittance status, it was found that studies with purely outpatient populations had, on average, higher EWB (weighted mean: 16.59 versus 13.30; P = 0.0434) and higher global QOL (weighted mean for total FACT-G score without RWD: 75.58 versus 62.60; P = 0.0071) when compared to studies with both outpatients and inpatients. After performing a literature search, no explanation to support these findings was found. We hypothesize that our results reflect the improved performance of outpatients when compared to inpatients.

A better EWB subscale score was recorded for studies which had > 30% of patients completing post-secondary education (mean: 17.66 versus 15.10; P = 0.0398) as well as a greater total FACT-G score without RWD (mean: 78.49 versus 69.60; P = 0.0477) when compared to cohorts with ≤ 30% completing post-secondary schooling. The correlation between levels of education and QOL has previously been analyzed [41]. Ross et al. hypothesized that since education gives access to nonalienated work and eventually wealth, it increases the sense of personal control [41]. In addition, education gives access to stable social relationships which increases social support [41]. Following this logic, it is clear why more educated cohorts had higher global QOL and EWB. Although RWD scores in cohorts with a higher incidence of previous radiation therapy reached statistical significance, no description explaining this correlation exists.

The present study is not without limitations. A first limitation is the cut-off ranges for cohort groupings may seem arbitrary. We have established the cut-off ranges in this way not only because they represent clear groupings by which studies in the literature are arranged, but also facilitate comparable sample sizes among groups. In addition, due to the many study arms included in the project (n = 39), more unbiased and therefore more noteworthy results may be presented. Second, the review is dominated by studies from the United States (n = 15; 51.5%) reflecting the high quantity of FACT-G QOL research conducted in this country. A number of patients with early stage disease were considered in the analysis because included cohorts were extensively heterogeneous with respect to disease progression. Additionally, the conclusions of the present study may or may not hold when analyses are conducted using patient-level data. A final limitation may be that the RWD and total FACT-G scores with RWD subscale analyses contain relatively small sample sizes.

Future work in either expanding on our results or in conducting similar analyses remains. As the FACT-G is not the only reliable QOL tool used in cancer patients, an interesting subject of analysis would be to perform a similar task with other general cancer questionnaires such as the EORTC QLQ-C30 to establish whether a discrepancy in findings exists. Other projects may aim to use the statistically significant correlations of the review and determine whether confounding parameters or cause and effect relationships exist.

## References

[1] World Health Organization. Cancer. 2012 Accessed from: http://www.who.int/mediacentre/factsheets/fs297/en/

[2] Gill TM, Feinstein AR (1994). A critical appraisal of the quality of quality-of-life measurements. JAMA.

[3] Chow E, Harris K, Fan G, Tsao M, Sze WM (2007). Palliative radiotherapy trials for bone metastases: a systematic review. J Clin Oncol.

[4] Cella DF, Tulsky DS, Gray G, Sarafian B, Linn E, Bonomi A, Silberman M (1993). The Functional Assessment of Cancer Therapy scale: development and validation of the general measure. J Clin Oncol.

[5] Luckett T, King MT, Butow PN, Oguchi M, Rankin N, Price MA, Hackl NA (2011). Choosing between the EORTC QLQ-C30 and FACT-G for measuring health-related quality of life in cancer clinical research: issues, evidence and recommendations. Ann Oncol.

[6] Wang Y, Shen J, Xu Y (2011). Symptoms and quality of life of advanced cancer patients at home: a cross-sectional study in Shanghai, China. Support Care Cancer.

[7] Esper P, Mo F, Chodak G, Sinner M, Cella D, Pienta KJ (1997). Measuring quality of life in men with prostate cancer using the functional assessment of cancer therapy-prostate instrument. Urology.

[8] Sharpe L, Butow P, Smith C, McConnell D, Clarke S (2005). Changes in quality of life in patients with advanced cancer: evidence of response shift and response restriction. J Psychosom Res.

[9] Ringash J, Lockwood G, O'Sullivan B, Warde P, Bayley A, Cummings B, Kim J (2008). Hyperfractionated, accelerated radiotherapy for locally advanced head and neck cancer: quality of life in a prospective phase I/II trial. Radiother Oncol.

[10] Crippa S, Dominguez I, Rodriguez JR, Razo O, Thayer SP, Ryan DP, Warshaw AL (2008). Quality of life in pancreatic cancer: analysis by stage and treatment. J Gastrointest Surg.

[11] Wakatsuki M, Tsuji H, Ishikawa H, Yanagi T, Kamada T, Nakano T, Suzuki H (2008). Quality of life in men treated with carbon ion therapy for prostate cancer. Int J Radiat Oncol Biol Phys.

[12] Kawahara M, Tada H, Tokoro A, Teramukai S, Origasa H, Kubota K, Shinkai T (2011). Quality-of-life evaluation for advanced non-small-cell lung cancer: a comparison between vinorelbine plus gemcitabine followed by docetaxel versus paclitaxel plus carboplatin regimens in a randomized trial: Japan Multinational Trial Organization LC00-03 (BRI LC03-01). BMC Cancer.

[13] Wong WS, Fielding R (2007). Quality of life and pain in Chinese lung cancer patients: Is optimism a moderator or mediator?. Qual Life Res.

[14] Marsland TA, Garfield DH, Khan MM, Look RM, Boehm KA, Asmar L (2005). Sequential versus concurrent paclitaxel and carboplatin for the treatment of advanced non-small cell lung cancer in elderly patients and patients with poor performance status: results of two Phase II, multicenter trials. Lung Cancer.

[15] Poon RT, Fan ST, Yu WC, Lam BK, Chan FY, Wong J (2001). A prospective longitudinal study of quality of life after resection of hepatocellular carcinoma. Arch Surg.

[16] List MA, Mumby P, Haraf D, Siston A, Mick R, MacCracken E, Vokes E (1997). Performance and quality of life outcome in patients completing concomitant chemoradiotherapy protocols for head and neck cancer. Qual Life Res.

[17] von Gruenigen VE, Frasure HE, Jenison EL, Hopkins MP, Gil KM (2006). Longitudinal assessment of quality of life and lifestyle in newly diagnosed ovarian cancer patients: the roles of surgery and chemotherapy. Gynecol Oncol.

[18] Zhou X, Cella D, Cameron D, Amonkar MM, Segreti A, Stein S, Walker M (2009). Lapatinib plus capecitabine versus capecitabine alone for HER2+ (ErbB2+) metastatic breast cancer: quality-of-life assessment. Breast Cancer Res Treat.

[19] Garland SN, Pelletier G, Lawe A, Biagioni BJ, Easaw J, Eliasziw M, Cella D (2011). Prospective evaluation of the reliability, validity, and minimally important difference of the functional assessment of cancer therapy-gastric (FACT-Ga) quality-of-life instrument. Cancer.

[20] Lo C, Burman D, Swami N, Gagliese L, Rodin G, Zimmermann C (2011). Validation of the QUAL-EC for assessing quality of life in patients with advanced cancer. Eur J Cancer.

[21] Garcia SF, Rosenbloom SK, Beaumont JL, Merkel D, Von Roenn JH, Rao D, Cella D (2012). Priority symptoms in advanced breast cancer: development and initial validation of the National Comprehensive Cancer Network-Functional Assessment of Cancer Therapy-Breast Cancer Symptom Index (NFBSI-16). Value Health.

[22] Bacik J, Mazumdar M, Murphy BA, Fairclough DL, Eremenco S, Mariani T, Motzer RJ (2004). The functional assessment of cancer therapy-BRM (FACT-BRM): a new tool for the assessment of quality of life in patients treated with biologic response modifiers. Qual Life Res.

[23] Cella DF, Bonomi AE, Lloyd SR, Tulsky DS, Kaplan E, Bonomi P (1995). Reliability and validity of the Functional Assessment of Cancer Therapy-Lung (FACT-L) quality of life instrument. Lung Cancer.

[24] Pandey M, Thomas BC, Ramdas K, Eremenco S, Nair MK (2004). Reliability & validity of the Malayalam Functional Assessment of Cancer Therapy for Head & Neck Cancer. Indian J Med Res.

[25] Yoo H, Suh C, Kim S, Eremenco S, Kim H (2006). Korean translation and validation of the functional assessment of cancer therapy-lung (FACT-L) version 4. Qual Life Res.

[26] Yount S, Cella D, Webster K, Heffernan N, Chang C, Odom L, van Gool R (2002). Assessment of patient-reported clinical outcome in pancreatic and other hepatobiliary cancers: the FACT Hepatobiliary Symptom Index. J Pain Symptom Manage.

[27] Yount S, List M, Du H, Yost K, Bode R, Brockstein B, Argiris A (2007). A randomized validation study comparing embedded versus extracted FACT Head and Neck Symptom Index scores. Qual Life Res.

[28] Pickard AS, Neary MP, Cella D (2007). Estimation of minimally important differences in EQ-5D utility and VAS scores in cancer. Health Qual Life Outcomes.

[29] Zimmermann C, Burman D, Swami N, Krzyzanowska MK, Leighl N, Moore M, Rodin G (2011). Determinants of quality of life in patients with advanced cancer. Support Care Cancer.

[30] Dharma-Wardene M, Au HJ, Hanson J, Dupere D, Hewitt J, Feeny D (2004). Baseline FACT-G score is a predictor of survival for advanced lung cancer. Qual Life Res.

[31] Martensson G, Carlsson M, Lampic C (2008). Do nurses and cancer patients agree on cancer patients' coping resources, emotional distress and quality of life?. Eur J Cancer Care (Engl).

[32] Hisamura K, Matsushima E, Nagai H, Mikami A (2011). Comparison of patient and family assessments of quality of life of terminally ill cancer patients in Japan. Psychooncology.

[33] Shaha M, Pandian V, Choti MA, Stotsky E, Herman JM, Khan Y, Libonati C (2010). Transitoriness in cancer patients: a cross-sectional survey of lung and gastrointestinal cancer patients. Support Care Cancer.

[34] Robson PC, Heffernan N, Gonen M, Thornton R, Brody LA, Holmes R, Brown KT (2010). Prospective study of outcomes after percutaneous biliary drainage for malignant biliary obstruction. Ann Surg Oncol.

[35] Lapitan MC, Buckley BS (2011). Impact of palliative urinary diversion by percutaneous nephrostomy drainage and ureteral stenting among patients with advanced cervical cancer and obstructive uropathy: a prospective cohort. J Obstet Gynaecol Res.

[36] Fumimoto H, Kobayashi K, Chang CH, Eremenco S, Fujiki Y, Uemura S, Ohashi Y (2001). Cross-cultural validation of an international questionnaire, the General Measure of the Functional Assessment of Cancer Therapy scale (FACT-G), for Japanese. Qual Life Res.

[37] Dapueto JJ, Francolino C, Servente L, Chang CH, Gotta I, Levin R, Abreu Mdel C (2003). Evaluation of the Functional Assessment of Cancer Therapy-General (FACT-G) Spanish Version 4 in South America: classic psychometric and item response theory analyses. Health Qual Life Outcomes.

[38] Cella D, Hahn EA, Dineen K (2002). Meaningful change in cancer-specific quality of life scores: differences between improvement and worsening. Qual Life Res.

[39] World Health Organization Department of Health Statistics. World Health Statistics 2011. World Health Organization 2011. Accessed from: http://www.who.int/whosis/whostat/EN_WHS2011_Full.pdf

[40] Poullier JP, Hernandez P, Kawabata K, Savedoff W http://www.who.int/healthinfo/paper51.pdf.

[41] Ross CE, Van Willigen M (1997). Education and the subjective quality of life. J Health Soc Behav.

